# Stable solution of induced modulation instability

**DOI:** 10.1038/s41598-020-66856-3

**Published:** 2020-06-22

**Authors:** Jingxin Guan, Zhanmei Ren, Qi Guo

**Affiliations:** Guangdong Provincial Key Laboratory of Nanophotonic Functional Materials and Devices, South China Normal University, Guangzhou, 510631 P. R. China

**Keywords:** Optics and photonics, Optical physics, Nonlinear optics

## Abstract

In this paper,we discussed the nonlinear evolution of modulation instability and the steady-state process of induced modulation instability in sine-oscillatory response nonlocal nonlinear media. With plane wave plus perturbation as initial conditions, we simulated the long-term evolution of modulation instability in the nonlocal nonlinear Schrodinger equation with sine-oscillatory response numerically. For the input of modulated wave, the approximate analytical solution of the stable solution of the equation is obtained under the assumption that only the fundamental wave and the first harmonic wave are present. For the input of modulated wave with arbitrary harmonic waves, we obtained the exact numerical solution of the stable solution of the induced modulation instability.

## Introduction

Modulation instability (MI) is considered as an important problem in nonlinear physics. A series of studies on MI have been carried out since 1960s, such as fluids^1^, plasma^2^, nonlinear optics^3–6^, among others. MI signifies the exponential growth of a weak perturbation of the amplitude of the wave as it propagates. The gain leads to amplification of sidebands, which breaks up the otherwise uniform wave front and generates fine localized structures. MI means the energy exchange between waves at different frequencies in essence, especially referring to the energy exchange between plane wave and perturbation at high-order harmonics^6^. The energy of plane wave is transformed into that of perturbation at high-order harmonics. Therefore, perturbation is amplified to lead to the generation of localized structures on plane wave during transmission. Theories have validated that perturbation can only trigger MI in a specific frequency range^3,4^. Reported was the first experimental observation of the modulational instability using single-mode fibers^5^. In nonlinear Kerr media, bright solitons can survive within the frequency range in which MI is generated; while dark soliton exist in the frequency range in which MI does not occur^4,7,8^. On the other hand, MI of a nonlinear system leads to the growth of amplitude of modulated wave when the modulated frequency of the input signals of the modulated wave within the frequency range in which MI is generated. The process is called induced modulation instability^3,9,10^. Hasegawa first proposed a theoretical scheme of generating ultra-high-rate pulse trains by utilizing induced modulation instability in fibers^3^, which has been experimentally verified by Tai *et al*.^10^.

A majority of researches on MI focus on discussing the linear process of MI during short-term transmission, that is processing the condition under small perturbation by using linear approximation method while ignoring the perturbation of high-order harmonics. However, the increment of perturbation is not much lower than the amplitude of plane wave after optical field evolves for a long term, so linear approximation method is not applicable. It is necessary to come up with new methods to discuss the nonlinear process when the increment of perturbation is not small after perturbation evolving for a long term. Beeckman first explored the nonlinear process of induced modulation instability in nonlocal nonlinear Kerr media during long-term evolution by using analytical and numerical methods^11^. Beeckman mainly discussed the nonlinear process of induced modulation instability in Kerr media with Gaussian response function^12–16^ and exponent response function^17–19^ and found that the evolution of optical fields exhibited quasiperiodicity in the process.

Sine-oscillatory response function is also a common one apart from Gaussian and exponent response functions of nonlocal nonlinear Kerr media, which was first put forward by Nikolov during the discussion of quadratic soliton^20^. Recently, researches have shown that it is feasible to attain a sine-oscillatory response function when the boundary of the system satisfies specific conditions in a bounded system^21^. Linear analysis has been carried out on MI in nonlocal nonlinear Kerr media with sine-oscillatory response^6^. In this paper, we discuss the long-term (nonlinear) evolution of the optical field under MI in nonlocal nonlinear Schrodinger equation with sine-oscillatory response through numerical method, and the characteristics of the evolution was also discussed. The stable solutions (approximate analytical solution and exact numerical solution) under induced modulation instability of nonlocal nonlinear Schrodinger equation with sine-oscillatory response was also found and the relationship between the beamwidth in the stable solution and two degrees of freedom (DOF) in the system was surveyed.

## Nonlinear evolutionary process of MI

The evolution of the linearly polarized electric field transmitted along *z*-axis in 1 + 1 dimensional media with sine-oscillatory response satisfies nonlocal nonlinear Schrödinger equation^6,21–23^

1 $$i\frac{\partial }{\partial z}u(x,z)+\frac{1}{2}\frac{{\partial }^{2}}{\partial {x}^{2}}u(x,z)-u(x,z)\,{\int }_{-\infty }^{+\infty }\,R(x-x{\prime} )|u(x{\prime} ,z){|}^{2}dx{\prime} =0,$$

where *u*(*x*, *z*) is the dimensionless slowly-varying complex amplitude of the optical field, *x* and *z* are, respectively, the dimensionless transverse coordinate and the dimensionless evolution coordinate, and the sine-oscillatory response function is expressed as follows^20,21^

2 $$R(x)=\frac{1}{2{w}_{m}}\text{sin}\left(\frac{|x|}{{w}_{m}}\right)$$

with *w*_*m*_ being the characteristic length of the nonlinear response. Mathematically, the *z* coordinate stand for time in equation above^24,25^, but it is, physically, the longitudinal space (beam-propagation direction) coordinate for the problem of the optical-beam-propagation^26–28^.

Previous research has been conducted on MI in nonlocal nonlinear Kerr media with sine-oscillatory response and mainly discusses the linear process of MI^6^. However, linear approximation method is not suitable owing to MI has been nonlinear when the optical field undergoes long-term evolution and the growing increment of perturbation cannot be ignored. Therefore, the nonlinear process of MI during long-term evolution would be discussed by employing numerical method. By taking infinite plane wave with perturbation

3 $${u(x,z)|}_{z=0}=1+{10}^{-4}\,\text{cos}({k}_{x}x),$$

as the initial input of Eq. (1), where the first term, the second term and *k*_*x*_ represent plane wave, perturbation and frequency of perturbation, respectively.

According to the previous research result of linear analysis on MI, it can be seen that the MI in the system described by Eq. (1) appears in the range of $1 < {w}_{m}^{2}{k}_{x}^{2} < {(4{I}_{0}{w}_{m}^{2}-1/4)}^{1/2}+1/2$ (where *I*_0_ denotes the intensity of plane wave)^6^. The amplitude of the perturbation will grow in the exponential form *e*^*gz*^ in the initial evolution stage when the frequency of the perturbation is within the range, where *g* refers to the linear gain coefficient of perturbation. At different frequencies, $g$ is expressed as

4 $$g=Re\left\{2|{k}_{x}|\sqrt{-\frac{{I}_{0}}{1-{w}_{m}^{2}{k}_{x}^{2}}-\frac{{k}_{x}^{2}}{4}}\right\}.$$

Moreover, *g* is infinite under $|{k}_{x}|=1/{w}_{m}$ in the range in which MI occurs to the system, whose corresponding frequency is the singular point in the range; the corresponding frequency at $g=0$ is the cutoff point in the range in MI happens. It can be seen from Eq. (4) that a perturbation whose frequency is closer to the singular point shows a larger linear gain coefficient in Kerr media with sine-oscillatory response; by contrast, a perturbation whose frequency is closer to the cutoff frequency exhibits a lower linear gain coefficient.

**Figure 1:**
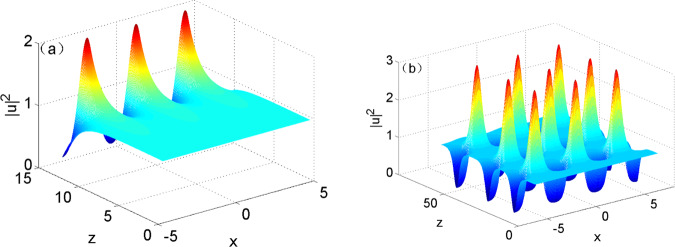
Simulated results in the evolutionary process of the optical field based on Eq. (1) with input condition (3) when ${w}_{m}=1$, ${k}_{x}=1.5$: (**a**) $z=12.6$; (**b**) $z=62.8$.

MI appears in the range of $1 < |{k}_{x}| < 1.61$ at ${w}_{m}=1$ and ${I}_{0}=1$; $|{k}_{x}|=1$ is the singular point in the range in which MI occurs, at which linear gain coefficient is infinite; and $|{k}_{x}|=1.61$ is the cutoff point in the range. By taking Eq. (3) as initial condition, the evolutionary process of the optical field under MI in media was simulated numerically, as shown in Fig. 1. In Fig. 1(a), it can be observed that the amplitude of perturbation exponentially grew in the short-term evolutionary process of the optical field, which validated the result of linear analysis on MI. However, it was impossible for perturbation to infinitely constantly increase. Linear approximation method was not applicable any more when the increment of perturbation grew to a point that cannot be ignored with the increase of evolution distance. It can be found from Fig. 1(b) that after growing in the initial evolution stage, perturbation started to attenuate and then evolved in a periodic-like oscillation form.

**Figure 2:**
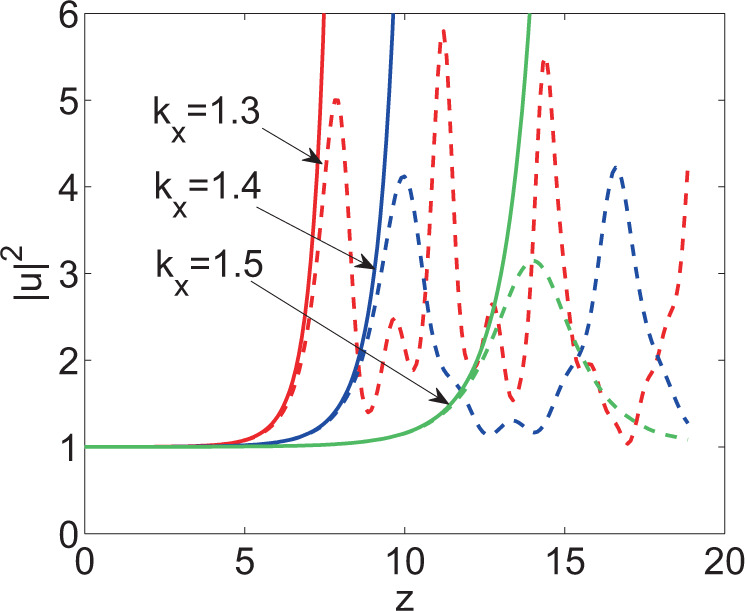
Evolution of the optical field based on Eq. (1) at $x=0$ under the input condition (3) when ${w}_{m}=1$ (solid line: analytical results obtained through linear approximation method; imaginary line: results attained through simulation).

A comparison was made between the analytical result in the evolutionary process of the optical field treated through linear approximation method and numerically simulated result without linear processing, as shown in Fig. 2. The solid and imaginary lines in the figure separately represent the analytical results and simulated results when inputting perturbation of different frequency. Linear approximation method was still applicable when perturbation was small in the short-term evolution of the optical field. In this case, the curve of intensity obtained through linear processing was favorably consistent with that attained through numerical simulation; linear approximation method was not applicable after the increment of perturbation grew to a large scale in the long-term evolution. Therefore, the evolution trend of the optical field obtained through linear approximation method gradually deviated from the simulated result. Additionally, it can be seen from Fig. 2 that the perturbation whose frequency was closer to the singular point exhibited faster increasing amplitude, more quickly reached the first peak and showed a larger peak; by contrast, the perturbation whose frequency was farther away from the singular point presented slower growth of the amplitude, reached the first peak later and attained a lower peak. It can be also seen in the Fig. 2 that the optical field was more irregularly evolved under the perturbation whose frequency was closer to the singular point.

**Figure 3:**
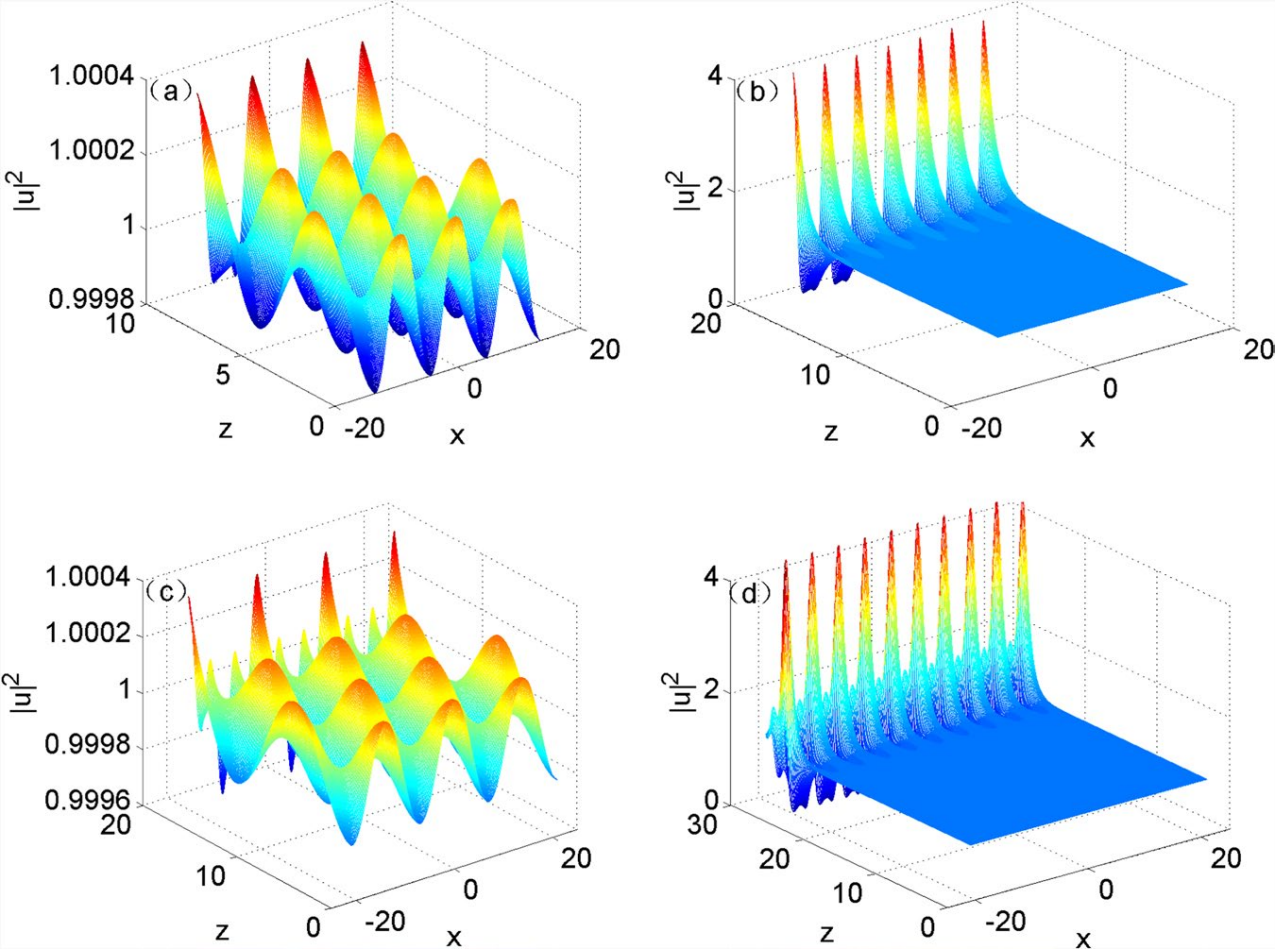
Evolution of the optical field based on Eq. (1) under input condition (3) when ${w}_{m}=1$: (**a**) ${k}_{x}=0.7$, $z=9.4$; (**b**) ${k}_{x}=0.7$, $z=18.8$; (**c**) ${k}_{x}=0.45$, $z=17.3$; (**d**) ${k}_{x}=0.45$, $z=28.3$.

Previous research indicated that perturbations would increase when their frequency fell in the range in which MI appeared; however, perturbations would be steadily transmitted and did not grow when their frequency was not found in the range^6^. It can be attained through numerical calculation that perturbations did not rise in the initial stage of short-term evolution of optical field when the frequency of perturbations was beyond the above range (Fig. 3(a,c)). In this case, optical field evolved in an oscillation form. However, high-harmonic generations (e.g. second- and third-harmonic generations) of perturbations were generated in the system after optical field was subjected to a long-term evolution. MI was also found when high-harmonic generation of perturbations appeared in the range of the system in which MI occurred (Fig. 3(b,d)). Figure 3(b,d) show MI separately induced by second- and third-harmonic generations of perturbations. On this basis, it can be seen that MI was also generated when high-harmonic generations of perturbations were found within the range in which MI appeared.

## Stable solution of induced modulation instability

### Approximate analytical solution of stable solution

To discuss the stable solution under induced modulation instability of Schrodinger equation in 1 + 1-dimensional media with sine-oscillatory response, it was necessary to introduce initial input $u(x,z){|}_{z=0}={\sum }_{n=-\infty }^{+\infty }{B}_{n}\,\text{exp}(i{k}_{x}nx)$ first (${B}_{n}$ was a constant). Based on the input, the solution to Eq. (1) is displayed as^11^

5 $$u(x,z)=\mathop{\sum }\limits_{n=-\infty }^{+\infty }\,{A}_{n}(z)\,\text{exp}(i{k}_{x}nx).$$

By substituting Eq. (5) into Eq. (1), it can be obtained

6 $$\begin{array}{c}i\,\mathop{\sum }\limits_{n=-\infty }^{+\infty }\,\frac{\partial }{\partial z}{A}_{n}(z)\,\text{exp}(i{k}_{x}nx)\\ \,-\,\frac{1}{2}\,\mathop{\sum }\limits_{n=-\infty }^{+\infty }\,{A}_{n}(z){k}_{x}^{2}{n}^{2}\,\text{exp}(i{k}_{x}nx)\\ \,-\,\mathop{\sum }\limits_{n=-\infty }^{+\infty }\,{A}_{n}(z)\,\text{exp}(i{k}_{x}nx)\,{\int }_{-\infty }^{+\infty }\,R(x-x{\prime} )[\mathop{\sum }\limits_{l=-\infty }^{+\infty }\,{I}_{l}(z)\,\text{exp}(i{k}_{x}lx{\prime} )]\\ \,dx{\prime} =0,\end{array}$$

where

7 $${I}_{l}(z)=\mathop{\sum }\limits_{q=-\infty }^{+\infty }\,{A}_{q}^{\ast }(z){A}_{l+q}(z).$$

Based on Eq. (6), various harmonic separately satisfy the following equation

8 $$i\frac{\partial }{\partial z}{A}_{n}(z)-\frac{1}{2}{A}_{n}(z){k}_{x}^{2}{n}^{2}-\mathop{\sum }\limits_{m=-\infty }^{+\infty }\,{A}_{m}(z){I}_{n-m}(z)\frac{1}{1-{w}_{m}^{2}{k}_{x}^{2}{(n-m)}^{2}}=0.$$

If Eq. (8) shows a stable solution, it means that the amplitudes of various harmonic in the optical field are a fixed value that does not vary with changing transmission distance; however, the change of phases is synchronous in the transmission process. Therefore, ${a}_{n}$ is expressed as ${A}_{n}={a}_{n}\,\text{exp}(i\beta z)$ and ${A}_{n}={A}_{-n}$^11^. By substituting ${A}_{n}={a}_{n}\,\text{exp}(i\beta z)$ into Eq. (8), it can be obtained

9 $$-\beta {a}_{n}-\frac{1}{2}{a}_{n}{k}_{x}^{2}{n}^{2}+s\,\mathop{\sum }\limits_{m=-\infty }^{+\infty }\,{a}_{m}{I}_{n-m}(z)\frac{1}{1-{w}_{m}^{2}{k}_{x}^{2}{(n-m)}^{2}}=0.$$

It is supposed that only plane wave and first harmonic are taken into account, i.e. only having ${a}_{0}$, ${a}_{1}$ and ${a}_{-1}$. According to Eq. (9), plane wave and first harmonic are separately obtained as

10 $$-\beta {a}_{0}-\left[{a}_{0}(|{a}_{0}{|}^{2}+2|{a}_{1}{|}^{2})+2\frac{{a}_{1}({a}_{0}^{\ast }{a}_{1}+{a}_{1}^{\ast }{a}_{0})}{1-{w}_{m}^{2}{k}_{x}^{2}}\right]=0;$$

11 $$-\beta {a}_{1}-\frac{1}{2}{a}_{1}{k}_{x}^{2}-\left[{a}_{1}(|{a}_{0}{|}^{2}+2|{a}_{1}{|}^{2})+\frac{{a}_{0}({a}_{0}^{\ast }{a}_{1}+{a}_{1}^{\ast }{a}_{0})}{1-{w}_{m}^{2}{k}_{x}^{2}}\right]=0.$$

Based on Eqs. (10) and (11), we can obtain

12 $$\begin{array}{c}{a}_{1}\left[{a}_{0}(|{a}_{0}{|}^{2}+2|{a}_{1}{|}^{2})+\frac{2{a}_{1}({a}_{0}^{\ast }{a}_{1}+{a}_{1}^{\ast }{a}_{0})}{1-{w}_{m}^{2}{k}_{x}^{2}}\right]\\ \,=\,\frac{1}{2}{a}_{0}{a}_{1}{k}_{x}^{2}+{a}_{0}\left[{a}_{1}(|{a}_{0}{|}^{2}+2|{a}_{1}{|}^{2})+\frac{{a}_{0}({a}_{0}^{\ast }{a}_{1}+{a}_{1}^{\ast }{a}_{0})}{1-{w}_{m}^{2}{k}_{x}^{2}}\right].\end{array}$$

On condition of introducing normalizing condition $|{a}_{0}{|}^{2}+2|{a}_{1}{|}^{2}=1$, by simplifying Eq. (12), we can attain

13 $$\frac{{a}_{1}^{2}}{{a}_{0}^{2}}=\frac{\frac{2}{1-{w}_{m}^{2}{k}_{x}^{2}}+\frac{1}{2}{k}_{x}^{2}}{\frac{4}{1-{w}_{m}^{2}{k}_{x}^{2}}-{k}_{x}^{2}},$$

Moreover, the intensity of plane wave in the optical field is

14 $${a}_{0}^{2}=\frac{\left(\frac{1}{1-{w}_{m}^{2}{k}_{x}^{2}}-\frac{{k}_{x}^{2}}{4}\right)(1-{w}_{m}^{2}{k}_{x}^{2})}{2},$$

The intensity of first harmonic is

15 $${a}_{1}^{2}=\frac{\left(\frac{1}{1-{w}_{m}^{2}{k}_{x}^{2}}+\frac{{k}_{x}^{2}}{4}\right)(1-{w}_{m}^{2}{k}_{x}^{2})}{4}.$$

By substituting Eqs. (14) and (15) into Eq. (10), the propagation constant of the optical field is attained as

16 $$\beta =-\,\frac{1}{1-{w}_{m}^{2}{k}_{x}^{2}}-1-\frac{{k}_{x}^{2}}{4}.$$

When only considering plane wave and first harmonic in the evolutionary process of the optical field, the approximate analytical solution of stable solution can be obtained as

17 $$\begin{array}{rcl}u(x,z) & = & [\sqrt{\frac{\left(\frac{1}{1-{w}_{m}^{2}{k}_{x}^{2}}-\frac{{k}_{x}^{2}}{4}\right)(1-{w}_{m}^{2}{k}_{x}^{2})}{2}}\\  &  & +\,\frac{\sqrt{\left(\frac{1}{1-{w}_{m}^{2}{k}_{x}^{2}}+\frac{{k}_{x}^{2}}{4}\right)(1-{w}_{m}^{2}{k}_{x}^{2})}}{4}\text{cos}({k}_{x}x)]\\  &  & \times \,\text{exp}\left[-i\left(\frac{1}{1-{w}_{m}^{2}{k}_{x}^{2}}+1+\frac{{k}_{x}^{2}}{4}\right)z\right].\end{array}$$

**Figure 4:**
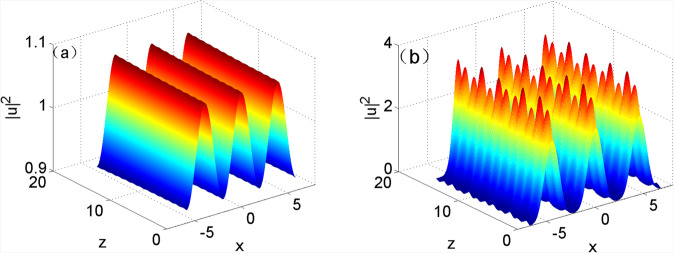
Approximate analytical solutions during the evolution of the optical field when ${w}_{m}=1$: (**a**) ${k}_{x}=1.6$; (**b**) ${k}_{x}=1.4$.

Simulation was carried out by taking the result of approximate analytical solution obtained using Eq. (17) at $z=0$ as the initial input of Eq. (1). The evolution of the optical field based on the equation under the input condition is displayed in Fig. 4. As shown in the figure, not all approximate analytical solutions at any modulation frequency can be steadily transmitted and only the approximate analytical solutions at the modulation frequency approximate to the cutoff frequency are able to be steadily transmitted (Fig. 4(a)); by contrast, the approximate analytical solutions at the modulation frequency greatly departing from the cutoff frequency greatly fluctuated in the transmission process (Fig. 4(b)). It indicated that the approximate analytical solution obtained on condition of only considering plane wave and first harmonic was applicable when modulation frequency approached to the cutoff frequency, while was not suitable when modulation frequency greatly departed from the cutoff frequency.

### Exact numerical solution of stable solution

The above approximate analytical solution was obtained in the case of only considering plane wave and first harmonic, and the approximation was suitable when the modulation frequency approached to the cutoff frequency. However, it was necessary to consider more number of harmonics during calculation when modulation frequency significantly departed from the cutoff frequency. Whereas, it was impossible to calculate the analytical solution when considering more enough harmonics, so the calculation was performed through numerical method. Based on normalizing condition

18 $$\mathop{\sum }\limits_{n=-{n}_{MAX}}^{{n}_{MAX}}\,|{a}_{n}{|}^{2}=1,$$

calculation was conducted on Eq. (9) by using Newton iteration method to calculate the values of ${a}_{n}$ and $\beta$^29^. By taking ${a}_{0}$, ${a}_{1}$, and $\beta$ in approximate analytical solutions as initial values, calculation was performed by constantly increasing the number of harmonics (that is increasing the number of equations) beginning with two harmonics. Iterative process was ended when harmonics with amplitude lower than 10^−4^ appeared. In this way, the numerical solution of stable solution can be obtained as

19 $$u(x,z)=\mathop{\sum }\limits_{n=-{n}_{MAX}}^{{n}_{MAX}}\,{a}_{n}\,\text{exp}(i\beta z)\,\text{exp}(i{k}_{x}nx).$$

The numerical solutions at all modulation frequencies were attained by utilizing the aforementioned numerical method. The amplitudes of each harmonic and propagation constant in the numerical solutions are shown in Table 1. Additionally, ${a}_{0}$, ${a}_{1}$ and $\beta$ in approximate analytical solutions obtained when only considering plane wave and first harmonic are also listed in Table 1.

**Table 1:** Amplitudes and propagation constants of each harmonic in numerical solutions at different modulation frequencies.

Modulation frequency *k*_*x*_	1.6	1.5	1.4	1.3	1.2	1.1
The number of harmonics in numerical solution	3	5	6	6	7	9
*a*_0_	0.9995 (0.9996)	0.9050 (0.9228)	0.8203 (0.8574)	0.7433 (0.8036)	0.6687 (0.7611)	0.5844 (0.7292)
*a*_1_	0.0220 (0.0200)	0.2992 (0.2724)	0.3978 (0.3639)	0.4553 (0.4209)	0.4849 (0.4587)	0.4850 (0.4839)
*a*_2_	0.0001	0.0317	0.0724	0.1264	0.1966	0.2807
*a*_3_	0.00002	0.0020	0.0078	0.0211	0.0508	0.1173
*a*_4_		0.0001	0.0007	0.0027	0.0097	0.0372
*a*_5_		0.00003	0.0001	0.0003	0.0016	0.0095
*a*_6_			0.00002	0.00001	0.0002	0.0021
*a*_7_					0.00001	0.0004
*a*_8_						0.0001
*a*_9_						0.00001
*β*	−0.9988 (−0.9990)	−0.7021 (−0.7625)	−0.2741 (−0.4483)	0.4409 (0.0268)	1.9065 (0.9127)	6.4225 (3.4594)

^1^Nonlinear characteristic length ${w}_{m}=1$, ${\sum }_{n=-{n}_{MAX}}^{{n}_{MAX}}|{a}_{n}{|}^{2}=1$.

^2^Data in the bracket refer to approximate analytical solutions.

It can be seen from Table 1 that the approximate analytical solution was favorably consistent with the numerical solution when modulation frequency approached to the cutoff frequency within the range in which MI appeared; however, there was a large difference between the two solutions when modulation frequency greatly away from the cutoff frequency. To validate whether the numerical solutions can be steadily transmitted, transmission was conducted by taking the numerical solutions as the initial input of Eq. (1), that is

20 $$u(x,z){|}_{z=0}=\mathop{\sum }\limits_{n=-{n}_{MAX}}^{{n}_{MAX}}\,{a}_{n}\,\text{exp}(i{k}_{x}nx).$$

**Figure 5:**
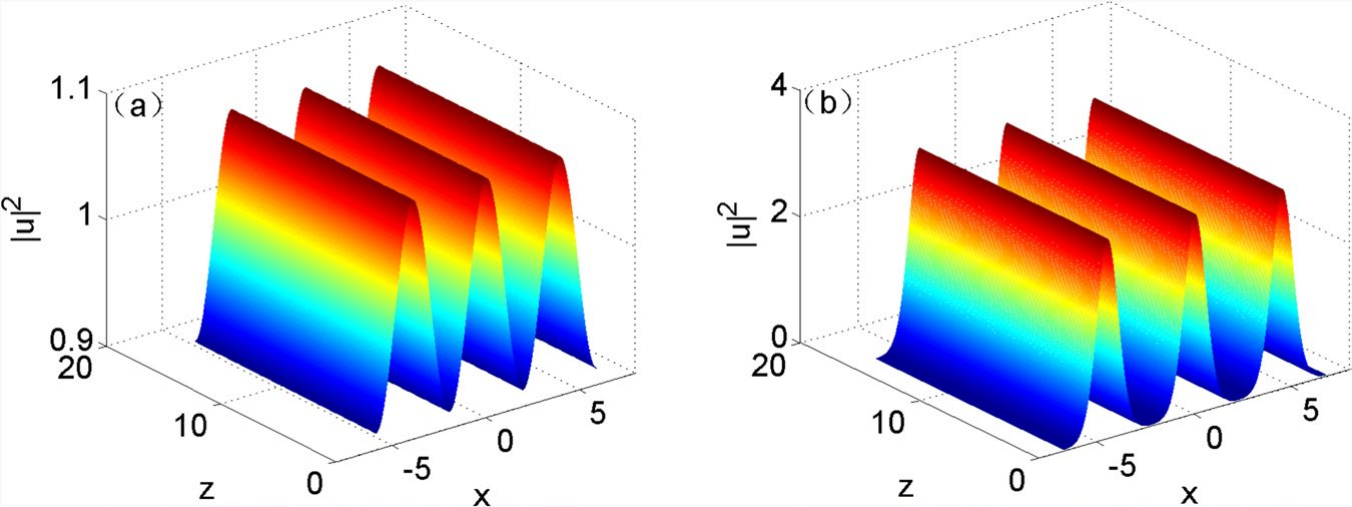
Numerical solution during the evolution of the optical field when ${w}_{m}=1$, (**a**) ${k}_{x}=1.6$; (**b**) ${k}_{x}=1.4$.

Figure 5 shows the simulated results of numerical solutions at different modulation frequencies in the evolution of the optical field. The figure revealed that the numerical solutions all can be steadily transmitted, which indicated that exact stable solutions can be attained by using the numerical method. By comparing Fig. 4(a) with 5(a), the approximate analytical solutions and numerical solutions all can be stably transmitted when the modulation frequency was approached to the cutoff frequency. It implied that the approximation method only considering plane wave and first harmonic was applicable in the case that the modulation frequency approached to the cutoff frequency; however, it was not suitable to the condition that the modulation frequency away from the cutoff frequency by comparing Fig. 4(b) with 5(b). It was also found from Table 1 that the difference between approximate analytical solutions and numerical solutions became increasingly larger, and therefore there were more harmonics in stable solutions when the modulation frequency more significantly departed from the cutoff frequency.

### Beamwidth of stable solution

According to Eq. (9), it can be obtained that there were $n$ equations for describing the system when considering $n$ harmonics and the equation set contained $n+3$ unknown quantities: amplitude ${a}_{0},{a}_{1},\ldots ,{a}_{n-1}$, nonlinear characteristic length ${w}_{m}$, propagation constant $\beta$ and modulation frequency ${k}_{x}$. Therefore, three DOFs were present in the system. Hence, three conditions {Relation (18) [that is, supposing the power of stable solution as $P=1$($P={\sum }_{n=-{n}_{MAX}}^{{n}_{MAX}}\,{a}_{n}^{2}$)], ${w}_{m}=1$ and ${k}_{x}$} were required when calculating stable solution in the section above (Exact numerical solution of stable solution). In this section, the characteristics of the stable solution with three DOFs was discussed. The beamwidth ${w}_{r}$ of stable solution within a single period was defined as

21 $${w}_{r}=\sqrt{\frac{2\,{\int }_{-\frac{T}{2}}^{\frac{T}{2}}\,{x}^{2}|u(x){|}^{2}dx}{{\int }_{-\frac{T}{2}}^{\frac{T}{2}}\,|u(x){|}^{2}dx}}$$

to characterize the stable solution and discuss the change relationships of ${w}_{r}$ with three DOFs ($P$, ${w}_{m}$ and ${k}_{x}$).

**Figure 6:**
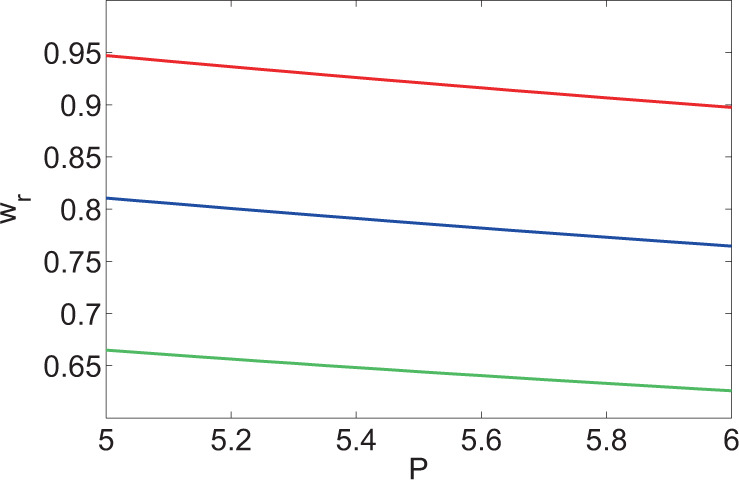
Curves of beamwidth of the stable solution with power under different values of ${w}_{m}$ in a system with sine-oscillatory response function (red line: ${w}_{m}=1$, blue line: ${w}_{m}=0.9$; green line: ${w}_{m}=0.8$).

At first, the change laws of ${w}_{r}$ in stable solution with the other two DOFs ${w}_{m}$ and $P$ were first explored on condition of keeping ${k}_{x}$ unchanged. The stable solution under different conditions were calculated through numerical method and the curve of ${w}_{r}$ with varying $P$ under different ${w}_{m}$ was attained, as shown in Fig. 6. As the range in which MI appeared was related to ${w}_{m}$, the selected values of ${w}_{m}$ in Fig. 6 were relatively approximated, so that the ranges in which MI appeared obtained under different values of ${w}_{m}$ showed a larger intersection (for convenience of comparison). As shown in the figure, ${w}_{r}$ decreased with increasing $P$ under same ${k}_{x}$ and ${w}_{m}$. The reason was that the cutoff frequency within the range in which MI occurred also grew with the increase of the power *P*^6^. It meant that ${k}_{x}$ would gradually depart from the cutoff frequency within the range in which MI happened with increasing $P$. Therefore, the number of harmonic in stable solution which needed to be considered rose, that is the spectral width of the stable solution grew, so that the beamwidth declined correspondingly. Similarly, the cutoff frequency also increased with the reduction of ${w}_{m}$ in the case of having same ${k}_{x}$ and $P$. In a similar way, the beamwidth was lowered.

Figure 6 displays the relationships of the beamwidth of the stable solution with power and nonlinear characteristic length at a given modulation frequency. The change law of beamwidth can be attained through scale transform at different modulation frequencies, instead of numerically calculating the equation again. Nonlocal nonlinear Schrodinger Eq. (1) exhibited transformation invariance, so the invariant transformation^23^ that Eq. (9) satisfied can be obtained

22 $${\bar{k}}_{x}=\kappa {k}_{x},\,{\bar{a}}_{n}=\kappa {a}_{n},\,{\bar{w}}_{m}=\frac{{w}_{m}}{\kappa },\,\bar{\beta }={\kappa }^{2}\beta .$$

It meant that Eq. (9) remained invariant via the transform (22). Therefore, the transformation relation of Σ_1_ and Σ_2_ in stable solution before and after transformation of the modulation frequency ${k}_{x}$ can be attained

23 $$\begin{array}{rcl}{\Sigma }_{1} & = & \{{k}_{x},{a}_{n},{w}_{m},\beta ,P,u(x,z),{w}_{r}\},\\ {\Sigma }_{2} & = & {\left\{{\bar{k}}_{x},\kappa {a}_{n},\frac{{w}_{m}}{\kappa },{\kappa }^{2}\beta ,{\kappa }^{2}P,\kappa u\left(\frac{x}{\kappa },\frac{z}{{\kappa }^{2}}\right),\frac{{w}_{r}}{\kappa }\right\}}_{\kappa =\frac{{\bar{k}}_{x}}{{k}_{x}}}.\end{array}$$

If the stable solution at a frequency ${k}_{x}$ had been calculated, the stable solution at any frequency ${\bar{k}}_{x}$ can be acquired through the transform (23).

**Figure 7:**
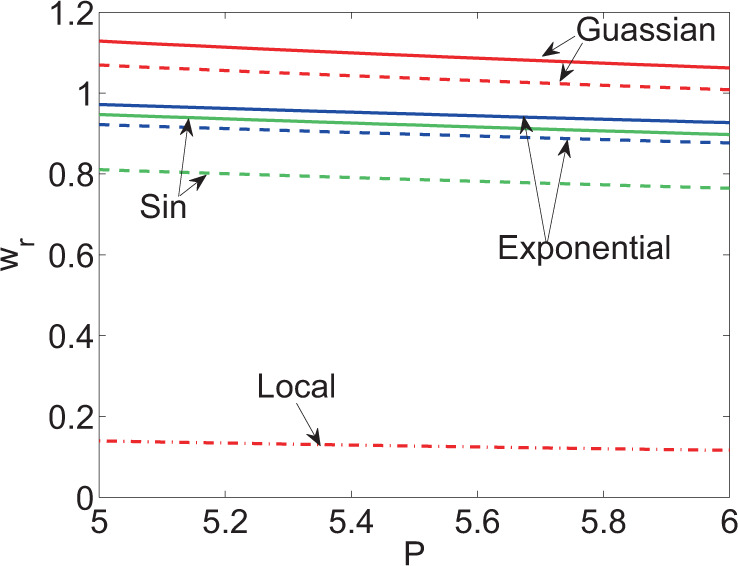
Curves of beamwidth of the stable solution with power under different values of ${w}_{m}$ in a system with different response functions (solid line: ${w}_{m}=1$, imaginary line: ${w}_{m}=0.9$ and dash-dotted line: local).

The stable solution of Schrodinger equation in nonlinear Kerr media with local, Gaussian and exponent responses have been found in some researches. Compared with the stable solution obtained in the study, the stable solution in these systems exhibited same characteristics, as shown in Fig. 7. The beamwidth ${w}_{r}$ of the stable solution decreased with increasing $P$ while rose with the growth of ${w}_{m}$ within a single period on the premise of keeping ${k}_{x}$ unchanged. Local response corresponded to special nonlocal response at nonlocal characteristic length ${w}_{m}=0$. Therefore, ${w}_{r}-P$ curve corresponding to nonlinear Kerr media with local response was found at the lowest part in Fig. 7.

## Conclusions

Two parts in a nonlocal nonlinear system with sine-oscillatory response were explored. On the one hand, it is nonlinear evolution of MI. The long-term evolution of optical field under MI of nonlocal nonlinear Schrodinger equation with sine-oscillatory response was numerically simulated by taking plane wave with perturbation as initial condition. Some characteristics in this process were obtained by simulating long-term nonlinear evolution of the optical field: firstly, perturbation started to attenuate after its amplitude grew to a certain value if its frequency was within the range in which MI occurred, and showing a quasi-periodic evolution in the long-term nonlinear process; secondly, perturbation whose frequency more approached to the singular point showed faster increasing amplitude, more quickly reached the first peak of its increment and exhibited a larger peak, moreover, the evolution of the optical field was increasingly irregular; thirdly, MI was also happened after long-term evolution if high-harmonic of perturbation were in the range in which MI appeared.

On the other hand, it is the steady-state process of induced modulation instability. The approximate analytical solution of stable solution under the induced modulation instability of nonlocal nonlinear equation with sine-oscillatory response was attained on condition of assuming only inputting plane wave and first harmonic (modulated wave); the exact numerical solution of stable solution was obtained when inputting the modulated wave with innumerable harmonics. By further discussing the stable solution, it can be found that fewer harmonics needed to be taken into account when calculating a stable solution if the modulation frequency of the stable solution was more approximated to the cutoff frequency in the range in which MI appeared. Additionally, the relationship of the beamwidth in the stable solution with two DOFs (power and nonlinear characteristic length) in the system were attained. On same conditions, the beamwidth reduced with increasing power while rose with the growth of nonlinear characteristic length.

## Methods

We used the split-step Fourier method^4^ to simulate the propagation of the optical beams. For the inputs Eqs. (3) and (20) at $z=0$, the evolution of Eq. (1) was obtained by the split-step Fourier method.

We used the Newton iteration method^29^ to obtain the exact numerical solution of stable solution, i.e. the solution of Eq. (9). By taking ${a}_{0}$, ${a}_{1}$, and $\beta$ in approximate analytical solutions as initial values, calculation was performed by the increase of the number of harmonics $n$ beginning with $n=0$, and $1$. Iterative process was ended when the amplitude of the last harmonics was lower than 10^−4^.
